# On-Farm Trials Reveal Significant but Uncertain Control of *Botrytis cinerea* by *Aureobasidium pullulans* and Potassium Bicarbonate in Organic Grapevines

**DOI:** 10.3389/fpls.2021.620786

**Published:** 2021-02-24

**Authors:** Anabelle Laurent, David Makowski, Nicolas Aveline, Séverine Dupin, Fernando E. Miguez

**Affiliations:** ^1^Department of Agronomy, Iowa State University, Ames, IA, United States; ^2^INRAE, UMR MIA 518, AgroParisTech INRAE, Université Paris-Saclay, Paris, France; ^3^Institut Français de la Vigne et du Vin–Vinopol̂e Bordeaux-Aquitaine, Blanquefort, France; ^4^Chambre d’Agriculture de la Gironde-Vinopôle Bordeaux-Aquitaine, Blanquefort, France

**Keywords:** Biopesticide, generalized linear multilevel model, Bayesian, *Botrytis cinerea*, on-farm trial, disease management

## Abstract

*Botrytis cinerea*, a fungal pathogen that causes gray mold on grapes, can decrease yield, substantially reduce wine quality, and therefore cause significant economic losses. In a context of increasing awareness of environmental and human health, biopesticides are a potential alternative to synthetic chemical treatments to produce grapes and wine in compliance with high food standards. However, the effectiveness of biopesticides is not well known and more research is needed to help winegrowers assess their ability to control wine diseases. Our study aims to assess the efficacy of two commercial biopesticides, based on potassium bicarbonate and *Aureobasidium pullulans*, in reducing the incidence of gray mold (i.e., the proportion of grape bunches that are diseased). We use data from an on-farm trial network managed over 3 years (from 2014 to 2016) in a major wine producing region located in Southwestern France, and fit Bayesian generalized linear multilevel models able to take the variability of treatment effect across trials into account. The fitted models were then used to estimate the efficacy on incidence as a function of the severity (i.e., the proportion of diseased grape berries in a bunch) in an untreated plot in order to determine if the effectiveness of the treatments depends on the disease pressure. At average disease severity (i.e., 3%), the efficacy on disease incidence at the network level was equal to 20% [95% CI = (−0.1; 37.3)] and 13% [95% CI = (0.2; 24.7)] for potassium bicarbonate and *A. pullulans*, respectively. For both biopesticides, the efficacy on incidence for a new site-year is highly uncertain, but potassium bicarbonate had a lower uncertainty and a lower application cost compared to *A. pullulans*. Our results confirm that potassium bicarbonate is an interesting biopesticide under farming conditions in organic vineyards in southwestern France, but the amount of uncertainty points to the need for further research.

## Introduction

Reducing the use of synthetic pesticides has become a major objective in Europe, particularly in France, where their use has remained at a high level for many years despite government actions (Hossard et al., 2017). On vines, synthetic pesticides are used several times a year to control various diseases caused mostly by pathogenic fungi (Chen et al., 2019). This crop accounts for around 14% of product purchases for less than 4% of the agricultural area occupied (Butault et al., 2011). The intensive use of synthetic pesticides has negative consequences on the environment by contaminating soil, surface and ground water (Komarek et al., 2009; Mailly et al., 2017), and on human health (Coleman et al., 2012; Chen et al., 2020).

In a context of increasing awareness of environmental and human health, there is a growing interest in using alternatives to synthetic pesticides to produce grapes and wine as they do not leave hazardous chemical residues in wine (Jacometti et al., 2010; Rotolo et al., 2018). Alternative methods can rely on the use of biopesticides based on macro/micro-organisms (also called biocontrol agents) and substances of natural origin such as plant and microbial extracts, mineral, and organic compounds (EPA, 2016; Nicot et al., 2016; Amichot et al., 2018). Biopesticides have been mostly evaluated under controlled conditions (Nicot, 2011; Haidar et al., 2016) and their performance under field conditions can be lower, or ineffective and highly variable, compared to controlled conditions, due to climatic variations, edaphic conditions, unstable quality of the product and difficulty in maintaining living organisms in good conditions (i.e., survival and colonization ability) (Nicot, 2011; Bardin et al., 2015; Pertot et al., 2017a). Thus, there is a need to test biopesticides under field conditions to assess their actual effectiveness and adapt control strategies against gray mold in vineyards (Nicot, 2011; Pertot et al., 2017a).

*Plasmopara viticola* (the causal agent of downy mildew), *Erysiphe necator* (the causal agent of powdery mildew), and *Botrytis cinerea* (hereafter called *B. cinerea*) account for the highest number of chemical treatments in several major vine producing regions, in particular in France. *B. cinerea*, a fungal pathogen that causes gray mold (also called botrytis bunch rot), can decrease yield, highly reduce wine quality, and cause significant economic losses (Jacometti et al., 2010). Botrytis bunch rot can mostly affect the plant around flowering time by causing inflorescences to dry and fall to the ground. Starting around bunch closure, gray mold can also affect the berries. The fungus will use the sugar in the berries to grow on the bunches and will destroy their organoleptic qualities. When the ripening starts, the berries become mostly insensitive to downy or powdery mildew. However, ripening is the period when gray mold expands the most and symptoms can appear until a few days before harvest. As the use of synthetic pesticides is not allowed at that time of the growing season, biopesticides represent an opportunity for winegrowers to control this disease late in the season.

Previous studies, conducted under field conditions (i.e., research vineyard or commercial vineyard), have reported mixed results on the efficacy of biopesticides against *B. cinerea* (Supplementary Table 1). Pertot et al. (2017b) evaluated anti-*B. cinerea* biopesticides with different mechanisms of action, alone and in mixtures, and showed that the average efficacy on disease severity (i.e., the percentage of reduction of diseased grapes berries using the treatment compared to the control) was high, with little variability between years and locations. Similar levels of efficacy were achieved with a single biopesticide and with multiple biopesticides. The experiments of Pertot et al. (2017b) were characterized by low levels of disease severity, and the use of management practices decreasing grape susceptibility (i.e., defoliation) which could explain the high efficacy levels obtained in these experiments. Other studies showed lower levels of efficacy. Rotolo et al. (2018) found that disease incidence (i.e., the percentage of reduction of diseased grapes bunches using the treatment compared to the control) was reduced by less than 30% under high disease pressure with a biopesticide treatment while the use of mixtures or the alternate use of biopesticides and synthetic fungicide (including fluopyram as an active ingredient) (Supplementary Table 1) showed efficacy up to 96%. They suggested combining biopesticides with limited use of synthetic fungicides as an alternative strategy to reduce the risk of fungicide resistance and residue levels. Calvo-Garrido et al. (2019) found that several commercial biopesticides products achieved 21–58% of efficacy in botrytis bunch rot severity, but not on every trial. Aziz et al. (2016) reported high reduction levels of disease incidence and on severity (up to 68 and 93%) with treatments combining several biopesticides (Supplementary Table 1). Youssef and Roberto (2014) studied the effect of salt solutions on *B. cinerea* on grapevine in field conditions. The efficacy on disease incidence ranged from 77 to 100% compared to the control, with potassium bicarbonate and potassium sorbate being the most effective salts. Fedele et al. (2020) showed a high, but variable, efficacy of *Aureobasidium pullulans* and *Trichoderma atroviride* in reducing *B. cinerea* colonization and sporulation on bunch trash associated with a reduction of diseased incidence compared to an untreated control. They also found that a single early season biopesticide application (at the full flowering stage) had a similar efficacy on *B. cinerea* colonization and sporulation on bunch trash than three applications at full flowering, pre-bunch closure, and veraison.

These studies provide useful information on the efficacy of different biopesticides but none of them attempted to relate the level of efficacy to the disease pressure. As biopesticide efficacy may depend on disease pressure, it would be valuable to establish a quantitative relationship between the effect of biopesticides on disease control and the disease pressure in the vineyard considered. Moreover, the results of past studies suggest that the effect of pesticides is highly variable between vineyards, but so far this variability has not been properly quantified. Finally, none of the previous studies relied on actual on-farm trials conducted by winegrowers using their own equipment in their own vineyards. Some of these studies were conducted in commercial vineyards but using non-commercial sprayer (i.e., small equipment spraying pesticides on grapes directly). To tackle these limitations, we assessed the efficacy of biopesticides on the incidence with the goal of understanding how the efficacy was affected by the disease pressure. We analyzed data from two on-farm research networks in organic production, based in Southwestern France, for which biopesticides on *B. cinerea* have been tested. In our analysis, disease severity in untreated vineyards was used as a proxy for disease pressure. All the data were collected in commercial vineyards conducted according to standard farming practices during 3 years in 23 and 26 sites-years, regarding the biopesticide. This dataset offers a unique opportunity to assess the efficacy of biopesticides under realistic conditions as winegrowers used commercially available field equipment and managed the on-farm trial with their standard management practices apart from the biopesticide treatment. Two biopesticides were considered, *A. pullulans* and potassium bicarbonate, and both were registered on the French market in 2012 and 2011, respectively. Their principles are very different as the first one is based on a living organism while the second is a non-biological product. Before 2011, two biopesticides, composed of *Bacillus subtilis*, were approved for organic production (Serenade^®^, and Serenade Max^®^), but the efficacy was low when disease pressure was relatively high, and the market price was high (110 €/ha) (RESAQVitiBio, 2015). In this context, the Vinopôle de Bordeaux-Aquitaine conducted several on-farm trials in order to assess the effectiveness of new products under farming conditions. Apart from the study conducted in one commercial vineyard divided in five trials by Youssef and Roberto (2014) over 2 years of experiment, our study is the first to report the results of trials assessing potassium bicarbonate in a network of field trials on vine against *B. cinerea*, composed of data coming from 23 trials from nine vineyards. Compared to our analysis, the study conducted by Youssef and Roberto (2014) is based on a smaller number of site-years, and did not analyze the uncertainty and variability of the treatment efficacy in detail. Our own analysis thus provides more insights about the efficacy of biopesticides to control *B. cinerea*.

Here, our objective is to evaluate whether the two above-mentioned biopesticides are effective in controlling the incidence of gray mold in commercial vineyards and whether their efficacy depends on the disease severity in untreated controls. We assessed the efficacy on incidence at the population-level (i.e., average across all trials) and for each trial separately in order to analyze the between-trial variability of the treatment efficacy. We also rigorously analyze the levels of uncertainty associated with our estimates using a Bayesian approach. Finally, we described the range of plausible levels of treatment efficacy for a new vineyard (out of sample) taking into account the observed variability of the treatment effect in our dataset.

## Materials and Methods

### Data Description and Experimental Design

The Vinopôle Bordeaux-Aquitaine managed an on-farm research network on the grapevine, called RESAQ VitiBio, to evaluate the efficacy of biopesticides against botrytis bunch rot in organic production. Two biopesticides, potassium bicarbonate (Armicarb^®^) and *A. pullulans* (Botector^®^) were tested in 23 and 26 trials, respectively, over 3 years (2014–2016) and nine locations per biopesticide. Each trial, defined as a unique combination of location and vintage (i.e., year of harvest), was composed of two replicates of two strips, one strip including untreated vines and the other including vines treated with one biopesticide. A pair of strips (biopesticide and the control) constitutes a replicate (i.e., block). Within each strip, four plots including 10 consecutive vine stocks (i.e., 5 m^2^) were used for measurements. Trials were distributed throughout Southwestern France, a major wine-producing area. Therefore, this study covers typical conditions of oceanic climate in this area with commonly used cultivars, management techniques and production targets (Supplementary Table 2). Winegrowers were all managing their vineyard with organic farming system. Armicarb^®^, is composed of 85% potassium bicarbonate and interacts directly with the pathogen by killing mycelium and spores while disrupting pH and osmotic pressure (Palmer et al., 1997). A first treatment was applied at veraison (change of berry color and accumulation of sugar, BBCH-scale 81), and up to two additional treatments were applied 2 weeks before harvest. These spraying dates were chosen according to manufacturer’s recommendations for both biopesticides. Botector^®^, is composed of two fungus strains of *A. pullulans*, DSM 14940 and DSM 14941, competing for nutrients and space with the pathogen (Elmer and Reglinski, 2006). Potassium bicarbonate was applied 1–3 times during ripening according to weather and risk of botrytis development. The use of *A. pullulans* required a mandatory application just before bunch closure (BBCH-scale 77) and up to two applications during ripening. During ripening, biopesticides were applied after heavy rainfall (>20 mm) or consecutive days of rain (3 mm per day) as it creates favorable conditions for the development of *B. cinerea* (Ciliberti et al., 2015). Applications were stopped 1 and 3 days before the harvest for potassium bicarbonate and *A. pullulans*, respectively. As a prophylactic method, some leaves were removed during the growing season for all the trials, as frequently done in organic systems (Weigle and Carroll, 2014).

At the end of each growing season, a few days before the cultivar-specific harvest date, Botrytis bunch rot incidence and severity were assessed by visually rating 50 grape bunches per plot. The incidence represents the percentage of bunches with symptoms (i.e., the number of bunches with symptoms divided by 50). The severity (also called intensity in the literature) represents the percentage of diseased berries (i.e., the sum of rotten berries per bunch divided by 50) and it is indicative of disease pressure. Disease severity is a variable with high interest as it reflects the impact on wine yield and gustative quality (Hill et al., 2010; Ky et al., 2012).

### Data Analysis

The biopesticides bicarbonate potassium and *A. pullulans* were assessed separately as they were not tested side-by-side. For each type of treatment, two statistical models, called GLRM_0 and GLRM_Int, were used:

The model GLRM_0, with trial and block as random effects, is defined as (Agresti, 2002; Makowski et al., 2014):

(1) $$Y_{i\,j\,k\,l}∼B\,e\,t\,a\,B\,i\,n\,o\,m\,i\,a\,l\,\left(n,\pi_{i\,j\,k},\phi \right)$$

(2) $$l\,o\,g\,i\,t\,\left(\pi_{i\,j\,k}\right)=\mu_{0}+\omega_{i}+\gamma_{j\,\left\{i\right\}}+\left(\mu_{T}+\theta_{i}+\tau_{j\,\left\{i\right\}}\right)\,X_{k}$$

$\omega_{i}∼𝒩\,\left(0,\sigma_{\omega }^{2}\right)$ idd

$\gamma_{j\,\left\{i\right\}}∼𝒩\,\left(0,\sigma_{\gamma }^{2}\right)$ idd

$\theta_{i}∼𝒩\,\left(0,\sigma_{\theta }^{2}\right)$ idd

$\tau_{i\,j\,k}∼𝒩\,\left(0,\sigma_{\tau }^{2}\right)$ idd

where *i* is the on-farm trial index (1, …, 23, or 26), *j* is the block index (1, 2), *k* is the treatment index (1 for control, 2 for biopesticide), *l* is the plot index (1, 2, 3, 4), *n* is equal to 50 and represents the number of bunches evaluated in the *i*-th on-farm trial, *j*-th block, *k*-th treatment for the *l*-th plot, π_*ijk*_ is the probability that a bunch is affected by botrytis in the *i*-th on-farm trial and the *j*-th block for the *k*-th treatment, *Y*_*ijkl*_ is the number of botrytis-affected bunches in a sample 50 bunches, ϕ is an overdispersion parameter (see section “Statistical Inference” below for more information), *X_k* is a binary variable equal to one if a biopesticide treatment was applied on the *l*-th sample in the *j*-th block of the *i*-th on-farm trial and equal to zero otherwise, *μ*_0_ is the mean value of the logit of π_*ijk*_ in untreated vineyard over all on-farm trials, *μ*_*T*_ is the mean biopesticide effect over on-farm trials, *ω*_*i*_ is the random trial effect, *γ*_*j*{*i*}_ is the random block effect nested within the *i*-th on-farm trial, *θ*_*i*_ is the random interaction between the *i*-th on-farm trial and the biopesticide effect, *τ*_*j*{*i*}_ is the random interaction between the *j*-th block nested with the *i*-th on-farm trial and the biopesticide effect.

According to this model, the global incidence (i.e., proportion of affected fruits based on mean parameter values) is expressed as $I_{0}=\frac{e^{\mu_{0}}}{1+e^{\mu_{0}}}$ without biopesticide and $I_{T}=\frac{e^{\mu_{0}+\mu_{T}}}{1+e^{\mu_{0}+\mu_{T}}}$ with biopesticide. The incidence in the *i*-th on-farm trial and block *j*-th is expressed as $I_{0\,i\,j}=\frac{e^{\mu_{0}+\omega_{i}+\gamma_{j\,\left\{i\right\}}}}{1+e^{\mu_{0}+\omega_{i}+\gamma_{j\,\left\{i\right\}}}}$ without biopesticide and $I_{T\,i\,j}=\frac{e^{\mu_{0}+\mu_{T}+\omega_{i}+\gamma_{j\,\left\{i\right\}}+\theta_{i}+\tau_{j\,\left\{i\right\}}}}{1+e^{\mu_{0}+\mu_{T}+\omega_{i}+\gamma_{j\,\left\{i\right\}}+\theta_{i}+\tau_{j\,\left\{i\right\}}}}$ with biopesticide.

Based on these quantities, several standard measures of treatment efficacy can be derived, both at the global average and block within trial levels. The risk ratio $\frac{I_{T}}{I_{0}}$ (or $\frac{I_{T\,i\,j}}{I_{0\,i\,j}}$ at the block within trial level) is expressed as the ratio of the proportion of affected fruits with biopesticide to the proportion of affected fruits without. The disease control efficacy on incidence (%) (hereafter called CE), expressed as $100\,\left(1-\frac{I_{T}}{I_{0}}\right),$ describes the percentage of reduction in incidence among treated bunches to untreated bunches. It is expressed as $100\,\left(1-\frac{I_{T\,i\,j}}{I_{0\,i\,j}}\right)$ for the block within trial *ij*. The odds ratio is another standard metric commonly used to assess treatment efficacy. The global Odds ratio is expressed as *e*^*μ*_*T*_^ and is equal to the ratio of the Odds of disease with and without biocontrol, i.e., $\frac{I_{T}}{1-I_{T}}/\frac{I_{0}}{1-I_{0}}$. The specific Odds ratio for the block within trial *ij* is equal to$\frac{I_{T\,i\,j}}{1-I_{T\,i\,j}}/\frac{I_{0\,i\,j}}{1-I_{0\,i\,j}}$. Risk ratio and Odds ratio lower than one indicates disease reduction with biocontrol.

The model (2) can be updated to take disease pressure (measured here by the severity at the control) into account. To do so, Eq. 2 should be replaced by

(3) $$l\,o\,g\,i\,t\,\left(\pi_{i\,j\,k}\right)=\mu_{0}+\beta_{1}\,Z_{j\,\left\{i\right\}}+\omega_{i}+\gamma_{j\,\left\{i\right\}}+\left(\mu_{T}+\theta_{i}+\tau_{j\,\left\{i\right\}}+\beta_{2}\,Z_{j\,\left\{i\right\}}\right)\,X_{k}$$

The model description remains the same as in (2) except for the variable *Z*_*j{i}*_ correspondingto the disease pressure measured by the severity (%) in the untreated control of the *j*-th block of the *i*-th on-farm trial. *β*_1_ is the regression parameter representing the effect of a 1% severity increase on the disease incidence in the control. *β*_2_ is a regression parameter for the interaction between the severity at the control and the biopesticide effect. With this model, the effect of a 1% severity increase on the disease incidence in the treated vine is equal to *β*_1_ + *β*_2_. With this model, it is also possible to measure the efficacy of the treatment through the computation of risk ratio, CE, and Odds ratio. However, with the model based on Eq. 3, these quantities are not constant but depend on the value of *Z*_*j{i}*_, i.e., on the disease pressure. Indeed, according to Eq. 3, t he proportion of affected fruits in the *i*-th on-farm trial and block *j*-th is expressed as $\frac{e^{\mu_{0}+\beta_{1}\,Z_{j\,\left\{i\right\}}+\omega_{i}+\gamma_{j\,\left\{i\right\}}}}{1+e^{\mu_{0}+\beta_{1}\,Z_{j\,\left\{i\right\}}+\omega_{i}+\gamma_{j\,\left\{i\right\}}}}$ without biocontrol and $\frac{e^{\mu_{0}+\mu_{T}+\beta_{1}\,Z_{j\,\left\{i\right\}}+\beta_{2}\,Z_{j\,\left\{i\right\}}+\omega_{i}+\gamma_{j\,\left\{i\right\}}+\theta_{i}+\tau_{j\,\left\{i\right\}}}}{1+e^{\mu_{0}+\mu_{T}+\beta_{1}\,Z_{j\,\left\{i\right\}}+\beta_{2}\,Z_{j\,\left\{i\right\}}+\omega_{i}+\gamma_{j\,\left\{i\right\}}+\theta_{i}+\tau_{j\,\left\{i\right\}}}}$ with biocontrol. The Odds ratio is then expressed as *e*^*μ*_*T*_ + *β*_2_*Z*_*j*{*i*}_ + *θ*_*i*_ + *τ*_*j*{*i*}_^ and, thus, decreases or increases as a function of *Z*_*j{i}*_ depending on the sign of *β*_2_.

### Statistical Inference

Models based on (2) and (3) are further denoted to as GLRM_0 and GLRM_Int. They were fitted using a Bayesian approach with the R package *brms* (Bürkner, 2017). The priors for the variance of the random effects were defined as a truncated Student’s *t* distribution with three degrees of freedom, zero lower bound and a 2.5 scale. The prior for the overdispersion parameter was defined as a gamma distribution, ϕ∼ Gamma(0.01,0.01). Posterior distributions for μ _0_, μ _*T*_, β 1, and *β*2 were computed using the No-U-Turn Samplers (Hoffmann and Gelman, 2014) as implemented in the Stan software (Stan Development Team, 2018). The convergence of the MCMC chains was checked with R packages *coda* and *brms* (Plummer et al., 2006; Bürkner, 2017). We ran four independent chains for 3,000 iterations and the metric *Rhat*, which compares the between- and within-chain estimates for model parameters, were between 1 and 1.02 which indicates convergence. Several other variants of GLRM were fitted and evaluated using the widely applicable information criterion (WAIC) values. The WAIC is a more general criterion than Akaike information criteria (Watanabe, 2013). As these alternative models showed a higher WAIC and less precise parameter estimates (i.e., wider credibility intervals), we did not select them. We also performed an efficient approximate Leave-one-out Cross-validation using the package *loo* (Vehtari et al., 2017), and computed concordance correlation coefficients (CCCs) using the package *agRee* (Feng, 2020) to assess and compare the candidate models. In particular, the overdispersion was found to be strong (Table 1), as frequently observed in plant disease epidemiology. Indeed, in many cases, Binomial models exhibit overdispersion with a variance much higher than the one predicted by this type of model (Bolker et al., 2009). Overdispersion can occur due to technical variation related to error measurements from the experimental design, missing covariates, an excess frequency of zero values, and biological variation between the subjects of interest. More information is given in Supplementary Tables 3, 4. Overdispersion can arise when models have been incorrectly specified by failing to include important predictors or by using other link functions (Richards, 2007; Harrison, 2014). Not accounting for overdispersion in a model can result in biased parameter estimates. One way to deal with overdispersion is to use the Beta-Binomial model (Harrison, 2015; McElreath, 2020), which includes an overdispersion term and assumes that each binomial count observation has its own probability of success.

**TABLE 1:** Widely applicable information criterion (WAIC), parameter estimates and 95% credible intervals for GLRM_0 and GLRM_Int for potassium bicarbonate and *Aureobasidium pullulans.*

Model	Parameter estimates and WAIC	Potassium bicarbonate	*Aureobasidium pullulans*
GLRM_0	WAIC	2,023	2,275
	μ_0_	−1.28 (−2.09; −0.48)	0.96 (−1.48; −0.44)
	μ_*T*_	−0.32 (−0.56; −0.07)	−0.17 (−0.35; −0.00)
	σ_*ω*_	1.85 (1.34; 2.62)	1.26 (0.93; 1.72)
	σ_*γ*_	0.19 (0.01; 0.41)	0.22 (0.02; 0.43)
	σ_*θ*_	0.23 (0.02; 0.48)	0.10 (0.01; 0.27)
	σ_*τ*_	0.27 (0.04; 0.46)	0.24 (0.07; 0.37)
	ϕ	18.96 (14.66; 24.11)	25.37 (19.71; 32.37)
GLRM_Int	WAIC	1,935	2,249
	μ_0_	−1.79 (−2.47; −1.12)	−1.24 (−1.71; −0.79)
	μ_T_	−0.16 (−0.47; 0.17)	0 (−0.23; 0.24)
	β 1	0.12 (0.09; 0.15)	0.11 (0.07; 0.16)
	β 2	−0.04 (−0.08; −0.01)	−0.06 (−0.12; −0.01)
	σ_*ω*_	1.51 (1.04; 2.17)	1.10 (0.80; 1.50)
	σ_*γ*_	0.21 (0.01; 0.44)	0.17 (0.01; 0.39)
	σ_*θ*_	0.30 (0.04; 0.56)	0.16 (0.01; 0.37)
	σ_*τ*_	0.32 (0.13; 0.50)	0.26 (0.11; 0.40)
	ϕ	30.33 (22.59; 40.26)	29.08 (22.06; 37.95)

At the on-farm network level, several outputs were computed from the samples representing posterior distributions, such as the posterior median and 95% credibility intervals of incidence under treated and untreated conditions, risk ratio, Odds ratio, and CE. The 95% credibility interval was directly computed from the samples drawn from the posterior distribution and used to describe uncertainty. We also computed 95% predictive intervals of the same quantities in order to describe plausible ranges of values for a new situation, similar to those included in our sample assuming similar conditions (Higgins et al., 2009; IntHout et al., 2016).

## Results

### Description of the Observed Proportions of Diseased Bunches

**FIGURE 1:**
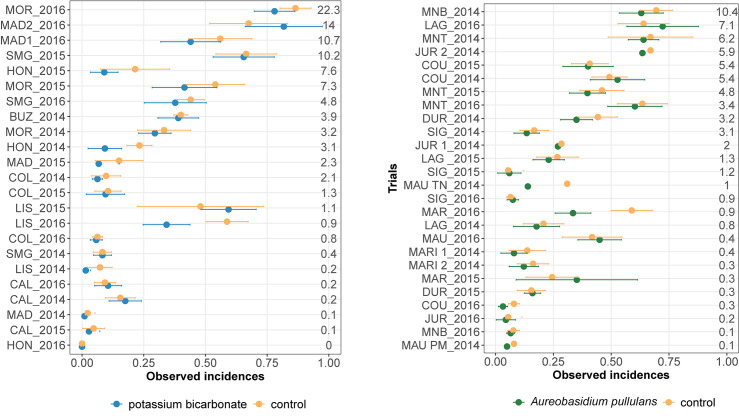
Observed disease incidences in treated bunches with potassium bicarbonate (blue dots, on the left) or with *Aureobasidium pullulans* (green dots, on the right) and in untreated grape bunches (yellow points) for each trial (site-year). Horizontal bars indicate 95% frequentist confidence intervals. Mean severity in the control is displayed for each trial on the right. Trials are ranked by increasing mean severity in the untreated control (%).

Observed mean disease incidences in treated and untreated bunches varied between trials for both biopesticides (Figure 1). The observed mean severity at the control per trial varied from 0 to 22% and from 0 to 10% for the on-farm network testing potassium bicarbonate and *A. pullulans*, respectively. About a third of the trials had an observed mean severity in the control lower than 5%.

Observed mean disease incidences in untreated bunches are generally higher than the mean disease incidence in treated bunches except for MAD2_2016, LIS_2015 (Figure 1, on the left), MAR_2015 and LAG_2016 (Figure 1, on the right). Most of the trials showed overlapping confidence intervals, between the biopesticide treatment and the untreated control, for the mean incidences (Figure 1). However, in LIS_2016 (Figure 1, on the left) and MAR_2016 (Figure 1, on the left), the mean incidence in untreated bunches was markedly higher than the disease incidence observed in treated bunches.

### Statistical Modeling Evaluation

**FIGURE 2:**
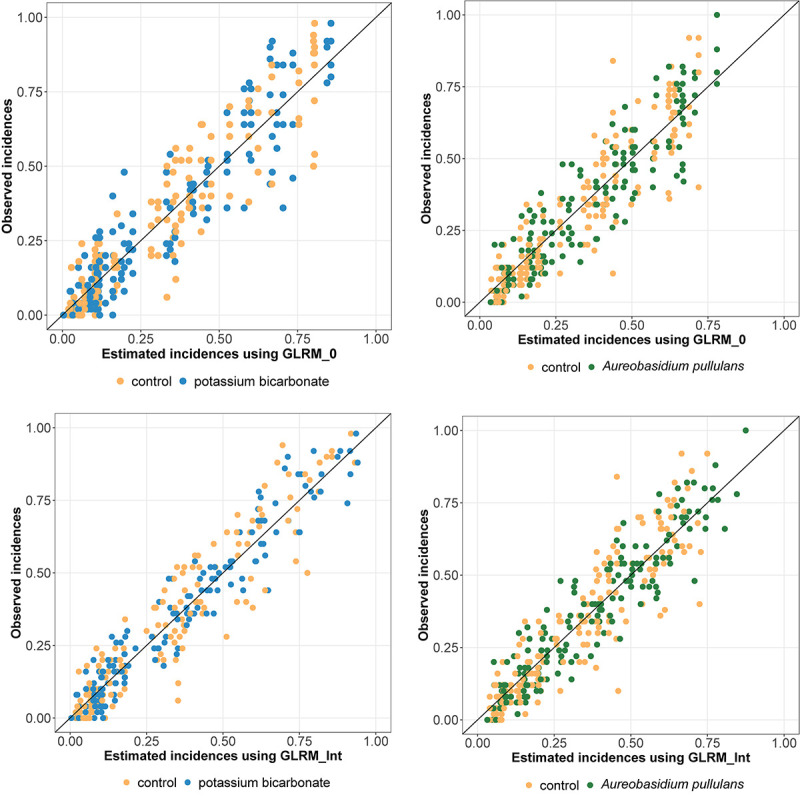
Observed vs. estimated incidences (posterior means) derived from the generalized linear multilevel model without (GLRM_0, at the top) and with disease pressure (GLRM_Int, at the bottom). Incidence for grape bunches treated with potassium bicarbonate or *Aureobasidium pullulans* are represented by blue or green dots, respectively. Incidence for untreated bunches are represented by yellow dots. One dot represents one replicate.

Regarding the WAIC values (Table 1), results were in favor of the model including disease pressure as a covariable (GLRM_Int) (i.e., GLRM_Int got a lower WAIC than GLRM_0). The Leave-one-out Cross-validation was in favor of GLRM_Int for both biopesticides. The graphical analysis of the observed vs. expected incidences derived from the posterior predictive distributions for GLRM_Int showed a better fit compared to GLRM_0 (Figure 2). The CCC was equal to 0.92 (0.90; 0.93) and 0.93 (0.91; 0.94) for GLRM_0 and GLRM_Int, respectively. The higher the CCC, the greater the concordance between the measured and the estimated incidences. Nevertheless, the model GLRM_0 remains relevant for estimating the global level of efficacy (averaged over all levels of disease pressure) of the biopesticides, especially because its WAIC is higher only by a narrow margin. The model GLRM_0 is useful for providing a general recommendation about the efficacy of the biopesticide across all values of disease pressure without the need of any field-specific observation of disease severity. On the other hand, GLRM_Int can be used to adjust treatment recommendations to specific values of disease pressure observed in vineyards.

### Estimated Efficacy of the Biopesticides

**FIGURE 3:**
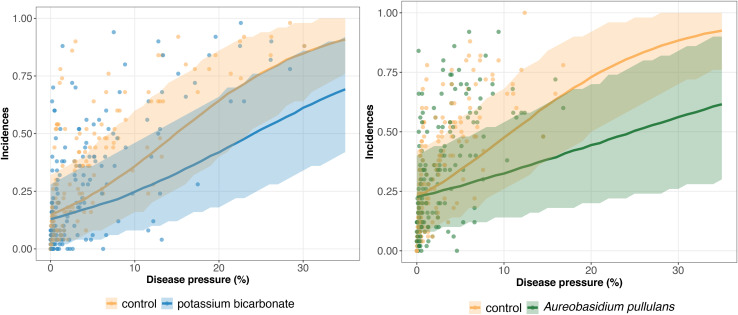
Estimated global disease incidence in bunches treated with potassium bicarbonate (blue line) or *Aureobasidium pullulans* (green line) and untreated grape bunches (yellow line) as a function of the disease pressure (disease severity in the untreated control) and their corresponding 95% credibility band (blue, green, and yellow curves and shadows). Blue and yellow dots represent the observed incidences in treated with potassium bicarbonate and untreated bunches, respectively. Green and yellow dots represent the observed incidences in treated with *Aureobasidium pullulans* and untreated bunches, respectively.

**FIGURE 4:**
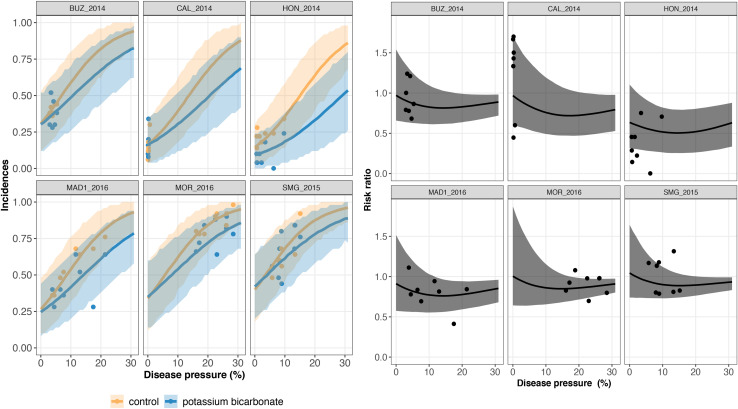
On the left, trial-specific estimated incidences in bunches treated with potassium bicarbonate (blue line) and untreated grape bunches (yellow line) as a function of the disease pressure (disease severity in the untreated control) and their corresponding 95% credible bands (blue and yellow shadows) for six different trials (out of 23). Blue and yellow dots represent the observed incidences in treated and untreated bunches, respectively. On the right, the corresponding estimated risk ratios (black line) with their 95% credible bands (gray shadow). Observed risk ratios are represented by black crosses and observed disease pressure levels are represented by black dots.

**FIGURE 5:**
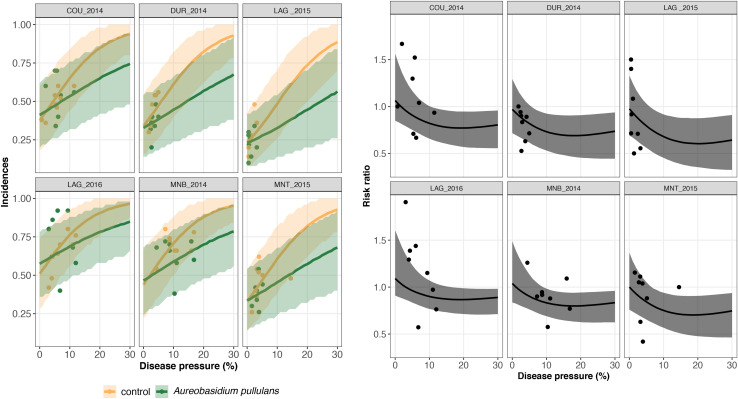
On the left, trial-specific estimated incidences in bunches treated with *Aureobasidium pullulans* (green line) and untreated grape bunches (yellow line) as a function of the disease pressure (disease severity in the untreated control) and their corresponding 95% credible bands (green and yellow shadows) for six different trials (out of 26). Green and yellow dots represent the observed incidences in treated and untreated bunches, respectively. On the right, the corresponding estimated risk ratios (black line) with their 95% credible bands (gray shadow). Observed risk ratios are represented by blue crosses and observed disease pressure levels are represented by black dots.

The results of model GLRM_Int revealed that global disease incidences (i.e., across all trials) increase as a function of the disease pressure (Figure 3). The estimated global disease incidences in untreated vineyards were higher in both networks regardless of the disease pressure level, although the uncertainty was large and credible intervals were overlapping (Figure 3). For low disease pressure, no substantial (i.e., small gap between the estimated global disease incidences with and without biopesticide, and overlapping credibility intervals) difference was found between the estimated disease incidences in the treated and untreated vineyards (Figure 3). For higher disease pressure, the gap between treated and untreated vineyards increased, showing a reduction in incidence when a biopesticide is applied. For disease severity levels between 0 and 10%, the incidence is lower when using potassium bicarbonate than *A. pullulans.* For example, when the severity at the control reaches 10%, the incidence with potassium bicarbonate is equal to 0.25 while the incidence with *A. pullulans* is equal to 0.32. There is a high heterogeneity in the measured incidences (dots on Figure 3) resulting from a strong between- and within-trial variability. The variability on the incidence between trials is illustrated on Figures 4, 5 for potassium bicarbonate and *A. pullulans*, respectively. To simplify the visualization of the results, only a subset of trials is presented but all the trials are displayed in Supplementary Figures 1–4. Each trial has different incidences as a function of the disease pressure. For example, at disease intensities of 10%, the estimated incidence in treated bunches with potassium bicarbonate were 18% in trial HON_2014 and 61% in trial SMG_2015 (Figure 4, on the left) while the global estimated incidence was equal to 25% (Figure 3 on the left). When the severity in the control was 10%, the estimated incidence in treated bunches with *A. pullulans* was 53% in trial COU_2014 and 44% in trial DUR_2014 (Figure 5, on the left), while the global estimated incidence was 32% (Figure 3 on the right).

A risk ratio lower than one indicates disease reduction with biocontrol. Above a threshold of 15–20% of severity, the individual risk ratio reached one as severity increased (Figures 4, 5). The 95% credible band of the risk ratio is wide when the observed incidences are widely spread (Figure 4 on the right, trial called CAL_2014) or when there is no observed values for a specific range of severity (Figure 4 on the right, trial called MOR_2016 for severity lower than 10%).

Odds ratio and risk ratio at the network level are in favor of potassium bicarbonate according to the results obtained with model GLRM_0 (Table 2). Based on model GLRM_Int, results are also in favor of potassium bicarbonate for low disease pressure, i.e., 2%. But for higher disease pressure levels, values tend to be in favor of *A. pullulans* but with large credible intervals revealing high uncertainty.

**TABLE 2:** Estimated values of risk ratio (RR) and odds ratio (OR) obtained withGLRM_0 and GLRM_Int (for three levels of disease severity in the control).

Model	Treatment efficacy	Estimated values (95% CI) for potassium bicarbonate	Estimated values (95%CI) for *A. pullulans*
GLRM_0	RR	**0.78 (0.63; 0.94)**	0.88 (0.77; 0.99)
	OR	**0.73 (0.57; 0.93)**	0.84 (0.71; 0.99)
GLRM_Int	RR 2%	**0.82 (0.64; 1.05)**	0.90 (0.79; 1.05)
	RR 10%	0.69 (0.52; 0.86)	**0.68 (0.51; 0.87)**
	RR 15%	0.65 (0.47; 0.84)	**0.62 (0.42; 0.86)**
	OR 2%	**0.79 (0.59; 1.06)**	0.88 (0.72; 1.07)
	OR 10%	0.58 (0.41; 0.80)	**0.53 (0.34; 0.80)**
	OR 15%	0.48 (0.29; 0.73)	**0.39 (0.19; 0.73)**

*Values in bold indicate the lowest between the two biopesticides considered.*

**FIGURE 6:**
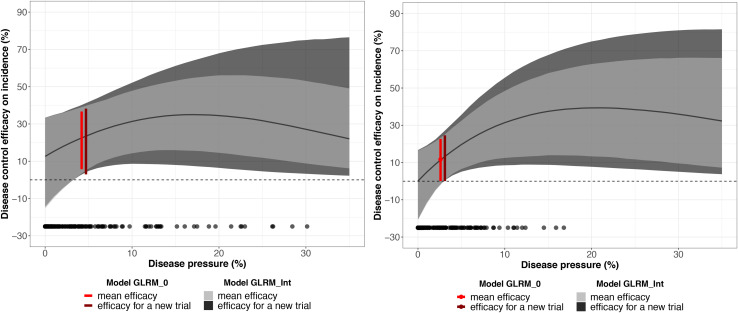
Mean disease control efficacy on incidence and its 95% credible interval (in light gray) estimated from the network testing the potassium bicarbonate (on the left) and *Aureobasidium pullulans* (on the right). The dark gray shadow represents 95% predictive intervals (plausible ranges for a new trial). The red vertical interval represents the mean disease control efficacy for a generalized linear multilevel model without covariate and its *x*-axis represents the disease pressure (disease severity in the untreated control). Black dots represent the observed disease pressure for all the replicates.

The global disease efficacy on incidence for bicarbonate potassium was twice as important as for *A. pullulans* according to the estimated values derived from GLRM_0 (Figure 6). The overall disease control efficacy on incidence was equal to 22.3 (5.7; 36.7) and to 11.7 (0.06; 22.7) for potassium bicarbonate and *A. pullulans*, respectively. With the GLRM_Int model, the global level of efficacy is higher with bicarbonate potassium than with *A. pullulans* for a disease pressure level between 0 and 10%. Above 10%, the global level of efficacy is higher with *A. pullulans*, but the uncertainty is large as most of the measured severity at the control is smaller than 10%. At 17 and 21% of severity, the global level of efficacy reached the highest value equal to 34.9 (15.6; 54.7) and 39.3% (13.2; 63.4) for potassium bicarbonate than *A. pullulans*, respectively, and then decreased. The uncertainty around the global level of efficacy is narrower for potassium bicarbonate as the range of observed severity is larger (from 0 to 31%) while the range of observed severity for *A. pullulans* was equal to 0–17%. Thus, for intensities higher than 17%, outputs for *A. pullulans* can be seen as an extrapolation and results should be interpreted with caution.

The efficacy for a new trial describes a plausible range of efficacy on incidence for a new vineyard (out-of-sample) conducted under similar conditions. For a severity at the control higher than 10%, the efficacy for a new trial is larger than the 95% credible interval at the network level, which reveals a substantial uncertainty regarding the efficiency of these biopesticides for a new trial.

## Discussion

In the present study, we assessed the efficacy on incidence of two biopesticides, *A. pullulans* and potassium bicarbonate, to an untreated control for the treatment of a grape disease (*B. cinerea*) through an on-farm research network managed using organic practices. Trials were different regarding the cultivars, rootstocks, densities, and locations used, and, thus, they area wide representation of practices for vineyards in France. Our aim was to perform a general evaluation and not simply a report of individual vineyard results. Our assessment confirmed that the data were better described by including the severity at the control as a covariable.

Our results are in favor of potassium bicarbonate regarding its efficacy on incidence at the network level, while considering that our data covered a wide range of disease pressures. Regarding the treatment application, potassium bicarbonate costs 45 €/ha and *A. pullulans* costs 80 €/ha for recommended doses (Caboulet et al., 2020). The predictive distribution for a new trial allows us to assess the heterogeneity and uncertainty in the treatment effect and represents a more appropriate treatment effect than the 95% credible interval at the population level.

Our results are consistent with those from Rotolo et al. (2018) as they found efficacies on incidence ranging from 8 to 23% for some biopesticides under high disease severity and, more specifically, the efficacy on incidence for *A. pullulans* was equal to 11%. The main difference is that 11 sprays were applied (off-label spray numbers for research purposes) while only 1–3 were applied in our trials. In another study conducted by Calvo-Garrido et al. (2019), the efficacy on incidence reached 18 and 17% for *A. pullulans* with five or six applications for a severity at the control equal to 17 and 13%, respectively. Our results do not agree with the study conducted by Pertot et al. (2017b) as an efficacy on incidence with *A. pullulans* was equal to 75%. This high value was explained by the relatively low-medium level of the disease and an optimal timing of application allowing *A. pullulans* to survive until harvest at concentrations sufficient to prevent *B. cinerea*. Comparisons against other studies should be interpreted with caution as they came from environments where indicators like temperature and the relative humidity differed, which impact the establishment of living organisms such as *A. pullulans* (Nicot, 2011).

Our results show a lower efficacy on incidence compared to the biopesticides combined mentioned in the literature (Supplementary Table 1) (O’Neill et al., 1996; Elmer et al., 2005; Cañamás et al., 2011; Pertot et al., 2017b) and this might suggest the presence of publication bias (Rothstein et al., 2005). Those experiments might have an advantage in terms of application timing while our trials were managed in real conditions by winegrowers who were having a first experience regarding the application of biopesticides. As a result, it is possible that the biopesticides were not applied in a timely fashion as farmers experienced lack of knowledge, technical or management issues, or unfavorable climatic conditions. As mentioned by Pertot et al. (2017b), the application timing is a crucial step, and *A. pullulans* should be applied at veraison, when sugar starts to increase, as it consumes the sugar needed for *B. cinerea* to grow and colonize grapevine wounds. The first application of potassium bicarbonate appears to be crucial, and if the fungus becomes visible, it will be difficult to control it (Wenneker and Kanne, 2009); thus, potassium bicarbonate should be used as a preventive method. Regarding synthetic chemical fungicides used against *B. cinerea*, Gabriolotto et al. (2009) compared the effectiveness of five different combinations of synthetic active ingredients including *boscalid*, *pyrimethanil*, *cyprodinil*, *fludioxonil*, *fenhexamid*, and *iprodione* to an untreated control in two commercial vineyards. Results showed an efficacy on disease incidence ranging from 23 to 73% with a mean of 56% using the synthetic active ingredients. Despite a high control of *B. cinerea* using synthetic chemical fungicides, there is a risk of resistance development and residue accumulation as mentioned by their analysis.

Our results covered a range of severity at the control before harvest from 0 to 17 and 0 to 30% for bicarbonate potassium and *A. pullulans*, respectively. Those values are consistent with real farming conditions in the study area and in previous studies where the values of severity ranged, all trials combined, between 4 and 20% (Reglinski et al., 2005; Cañamás et al., 2011; Aziz et al., 2016; Pertot et al., 2017b; Calvo-Garrido et al., 2019) except for Rotolo et al. (2018) who reported 31 and 53% of severity at the control before harvest in two different trials.

The winegrowers participating in this network use commercially available field equipment and standard management practices apart from the biopesticide treatment. Analyzing the trials from different vineyards together using a generalized linear multilevel model allowed us to understand the overall efficacy of the tested biopesticides besides the efficacy at the trial level. Also, the uncertainty was estimated to provide a range of plausible values of efficacy and help decision making about biopesticides. In addition to the overall efficacy, the efficacy for a new trial provides a more complete picture of the range of plausible effects of the tested biopesticides in a new environment (i.e., not initially included in the network of trials) (Laurent et al., 2020).

Our data showed a substantial between-trial variability (Table 1), which can be explained by the diversity of agronomic management, pedoclimatic conditions, sensitivity of grapes varieties to *B. cinerea*, vine density, and timing of application (Supplementary Table 2). The aim of the work is also to find a way to analyze heterogeneous data under a common framework and show that we can estimate efficacy values considering widely different environmental and management conditions.

Prior to the arrival of biopesticides on the market for controlling *B. cinerea*, organic vine producers only benefited from prophylactic methods (e.g., leaf or bunch removal). *B. cinerea* can lead to a strong qualitative depreciation on wines from only 5% of Botrytis-affected grapes that cause degradation of color, aroma, and structure (Ky et al., 2012), which reinforces the need to provide additional and reliable management practices for organic producers. Biopesticides represent a promising tool in Integrated Pest Management as they can be combined with chemical fungicides to reduce the risk of fungicide resistance and high residue levels. Indeed, the synthetic products can be applied early in the season (e.g., flowering, pre-closure, and veraison), while biopesticides can be planned for later before harvesting (Rotolo et al., 2018).

## Conclusion

In conclusion, the analysis of on-farm trial data confirmed a partial efficacy of bicarbonate potassium when the disease pressure does not exceed 20%. The efficacy on disease incidence for *A. pullulans* is lower and the uncertainty is relatively high. Compared to the inorganic compound potassium bicarbonate, *A. pullulans* is a living organism and needs to colonize and survive in an environment with fluctuating climatic conditions (Bardin et al., 2015) which can affect its efficacy. Cost applications are also in favor of the use of potassium bicarbonate. The biopesticide based on potassium bicarbonate represents an alternative management practice for controlling *B. cinerea* in organic grapevine production without synthetic pesticides. Indeed, potassium bicarbonate treatments are affordable, are easier to apply than living organisms (such as *A. pullulans*), and showed a reduction in the disease incidence between 13 and 32% over vineyards characterized by a disease severity of 0–20%. In the wine-growing area of our study, the interest for testing management practices through an on-farm research network is increasing, thus our proposed approach should be useful in future research efforts.

## Data Availability Statement

The datasets generated during and/or analyzed during the current study are owned by the Institut Français de la Vigne et du Vin–Vinopôle Bordeaux-Aquitaine. Please contact NA and SD for more information. Requests to access the datasets should be directed to NA, Nicolas.AVELINE@vignevin.com.

## Author Contributions

AL wrote the first draft of the manuscript and performed the statistical analysis and the visualization. NA and SD contributed in experimental work and organized the database. DM and FM contributed to the data analysis and supervised the work. All authors contributed to manuscript revision, read, and approved the submitted version.

## Conflict of Interest

The authors declare that the research was conducted in the absence of any commercial or financial relationships that could be construed as a potential conflict of interest.

## Abbreviations

CCC: concordance correlation coefficient
CE: control efficacy on incidence
GLRM: generalized linear random-effect model
WAIC: widely applicable information criterion.
