# Supplementary material for: On-Farm Trials Reveal Significant but Uncertain Control of Botrytis cinerea by Aureobasidium pullulans and Potassium Bicarbonate in Organic Grapevines
Source: Front Plant Sci. 2021 Feb 24;12:620786. doi: 10.3389/fpls.2021.620786 (PMC7943639; doi:10.3389/fpls.2021.620786)
Supplement: Supplementary file 1 [file Data_Sheet_1.PDF]

On-farm trials reveal significant but uncertain control of *Botrytis cinerea* by *Aureobasidium pullulans* and potassium bicarbonate in organic grapevines

Supplementary Material

Anabelle Laurent, David Makowski Nicolas Aveline, Séverine Dupin, Fernando Miguez

Required R packages and corresponding version

```
library(brms)     # version 2.13.0
# You do need to install the R package rstanarm to run brms
```

Table 2: Management and agronomic characteristics of the trials part of the network

Trial identifier*	Surface (ha)	Cultivar	Rootstocks Group	Density (vines per ha)	Pruning	Year of plantation	Particular sensibility
BUZ	0.5	Cabernet Franc	NA	4300	simple guyot	NA	Botrytis
CAL	0.6	Merlot	3309 C	5700	Bordeaux	1960	NA
COL	3	Sauvignon Blanc	SO4	2525	double guyot	1982	Botrytis
COU	3.3	Sauvignon Blanc	Gravesac	3300	simple guyot	2009	NA
DUR	0.8	Merlot	41-B	4500	simple guyot	1998	Botrytis
HON	NA	Folle Blanche	3309 C	3636	Cordon Royat	NA	Botrytis and downy mildew
JUR	0.2	Courbu & Camaralet	3309 C	2747	simple guyot	1998	NA
LAG	0.7	Merlot	3309 C	5750	mixed guyot	2001	NA
LIS	0.5	Merlot	NA	8300	Cordon Royat	2002	Botrytis and downy mildew
MAD	0.5	Tannat	3309 C	4938	simple guyot	2001	NA
MAR	3.8	Merlot	101-14,3309 C, Riparia Gloire de Montpellier	9000	double guyot	1997	NA
MAU1	0.8	Tannat	3309 C	4000	simple guyot	1979	Berry moth
MAU2	1.2	Tannat	3309 C	4000	simple guyot	1968	Botrytis

Trial identifier*	Surface (ha)	Cultivar	Rootstocks Group	Density (vines per ha)	Pruning	Year of plantation	Particular sensibility
MNB	0.7	Sauvignon Blanc	101-14	4166	mixed guyot	2006	Botrytis
MNT	2.4	Merlot	3309 C	6000	mixed guyot	1998	Botrytis and downy mildew
MOR	0.7	Muscadelle	101-14	2900	double guyot	1990	Botrytis
SIG	0.7	Sauvignon Blanc	101-14	4500	simple guyot	1989	Botrytis
SMG	1.3	Muscadelle	101-14	5000	simple guyot	2006	Botrytis

- The trial identifiers are the same as the ones used for the figures.

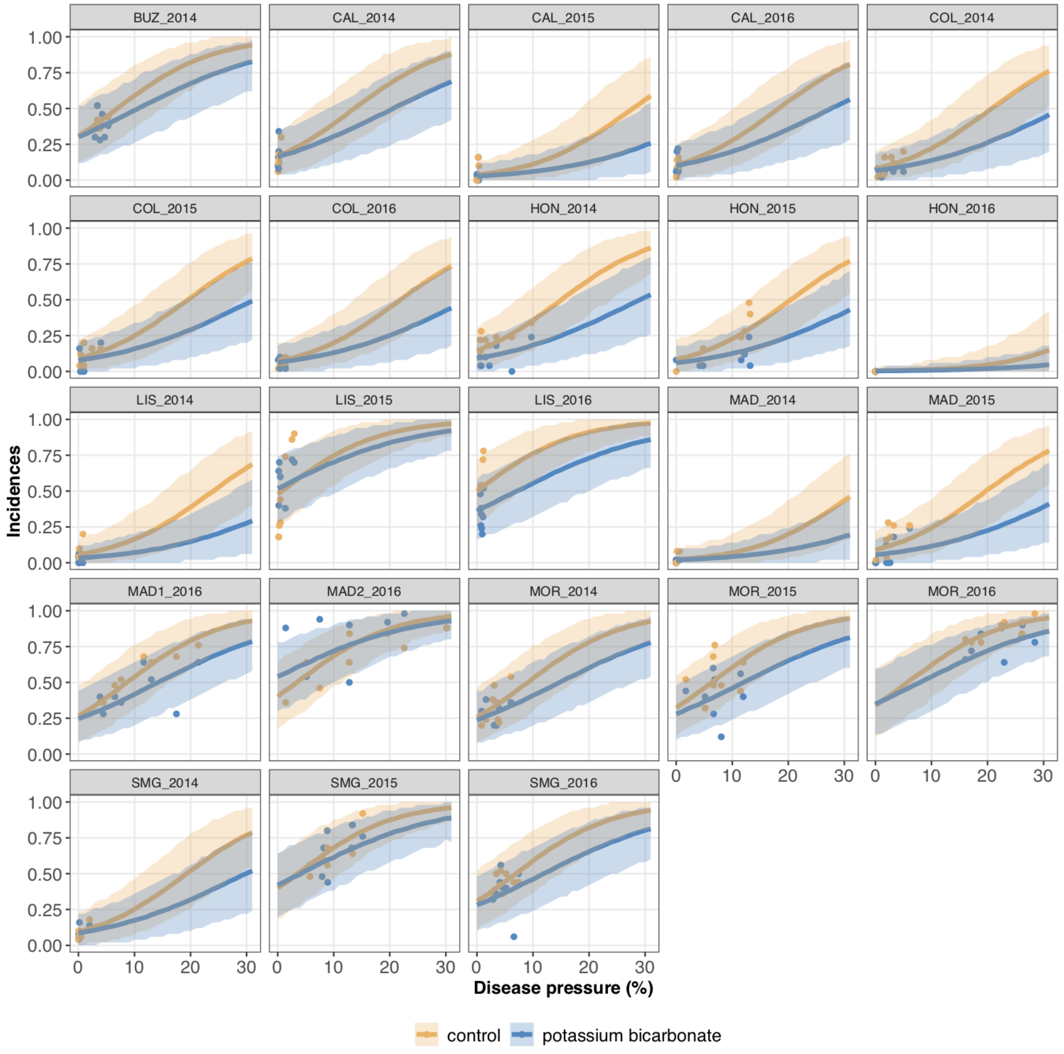

Figure 1: Individual incidence in treated bunches with potassium bicarbonate (blue line) and untreated bunches (yellow line) as a function of the intensity at the control and their corresponding 95% credible band

(blue and yellow shadows). Blue and yellow dots represent the observed incidences in treated and untreated bunches, respectively.

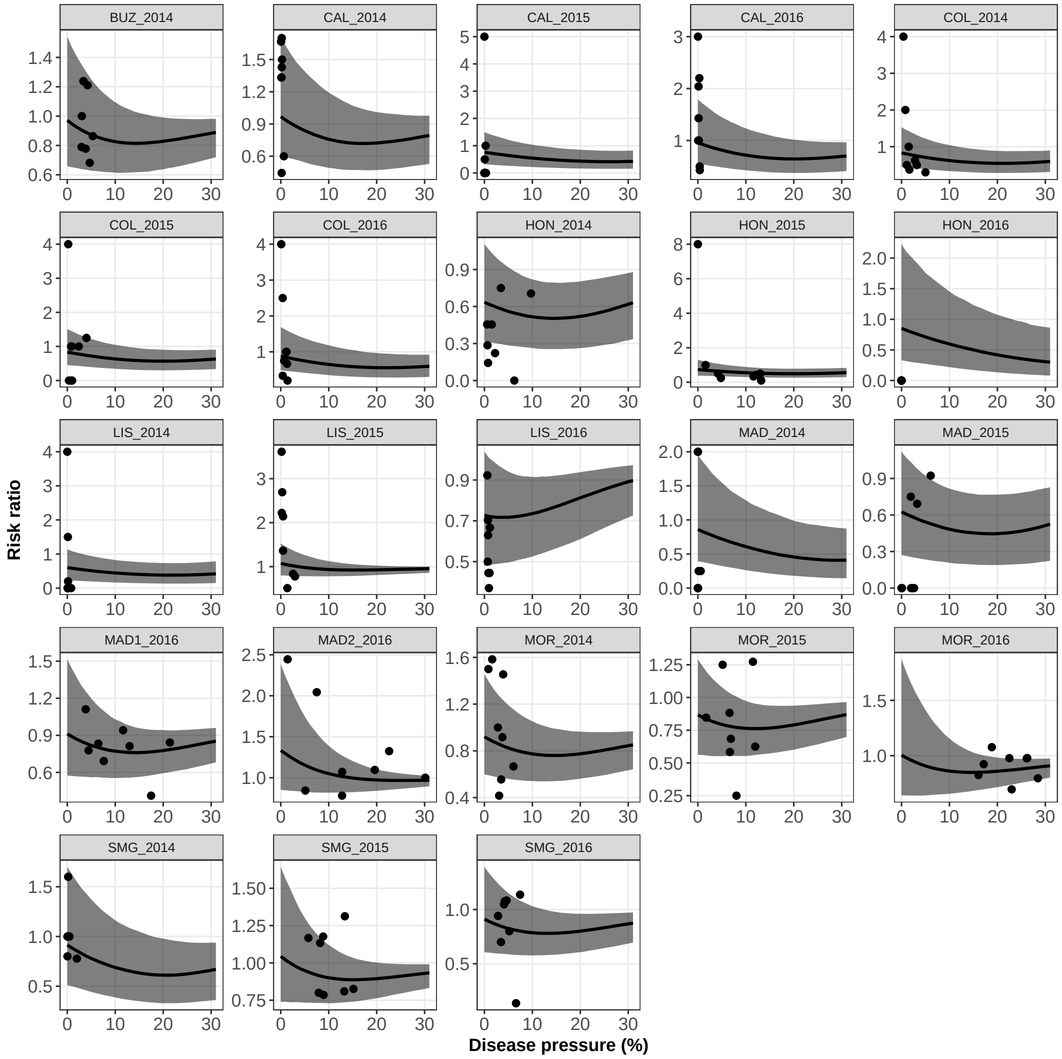

Figure 2: Individual risk ratios with potassium bicarbonate (black line) with their 95% credible bands (grey shadow) as a function of the intensity at the control. Observed risk ratio are represented by black dots.

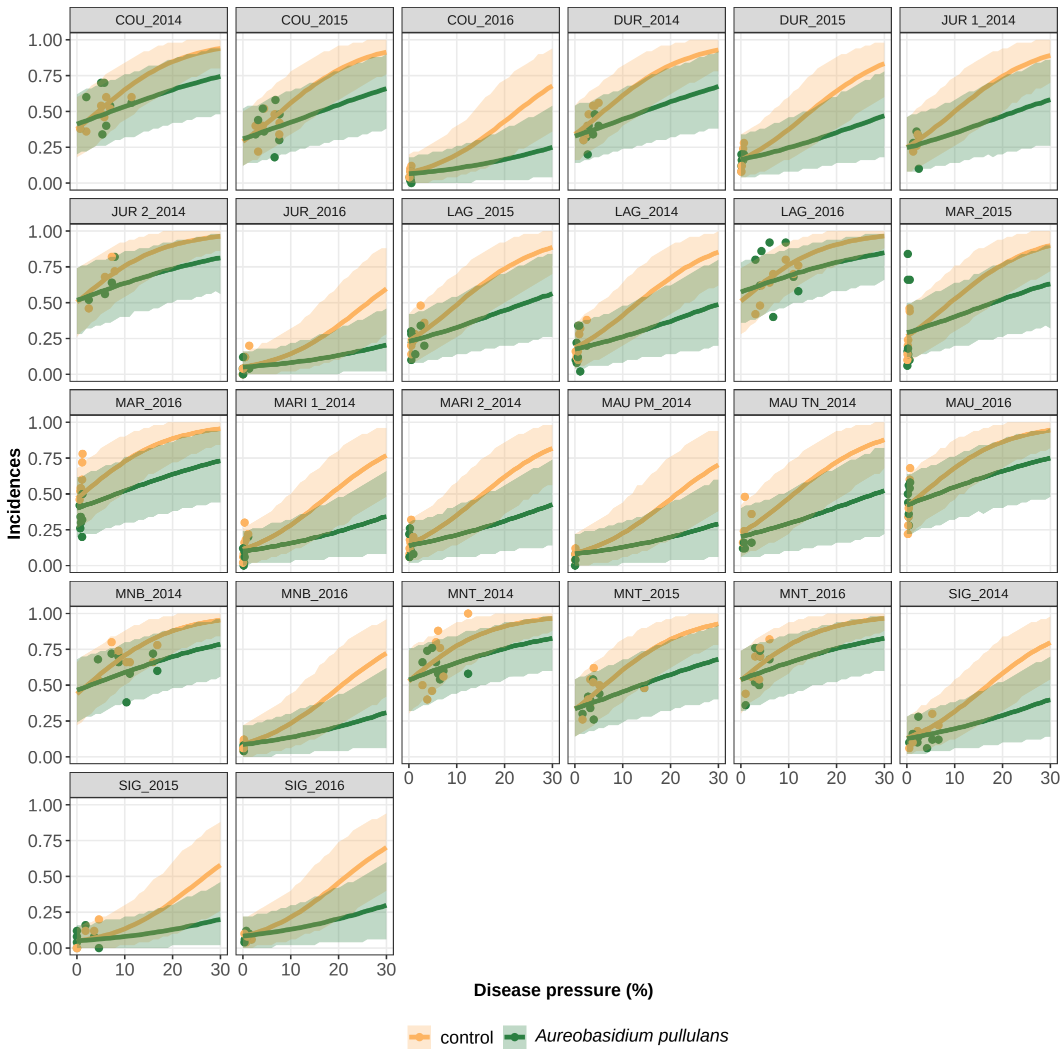

Figure 3: Individual incidence in treated bunches with *Aureobasidium pullulans* (green line) and untreated bunches (yellow line) as a function of the intensity at the control and their corresponding 95% credible band (green and yellow shadows). Green and yellow dots represent the observed incidences in treated and untreated bunches, respectively.

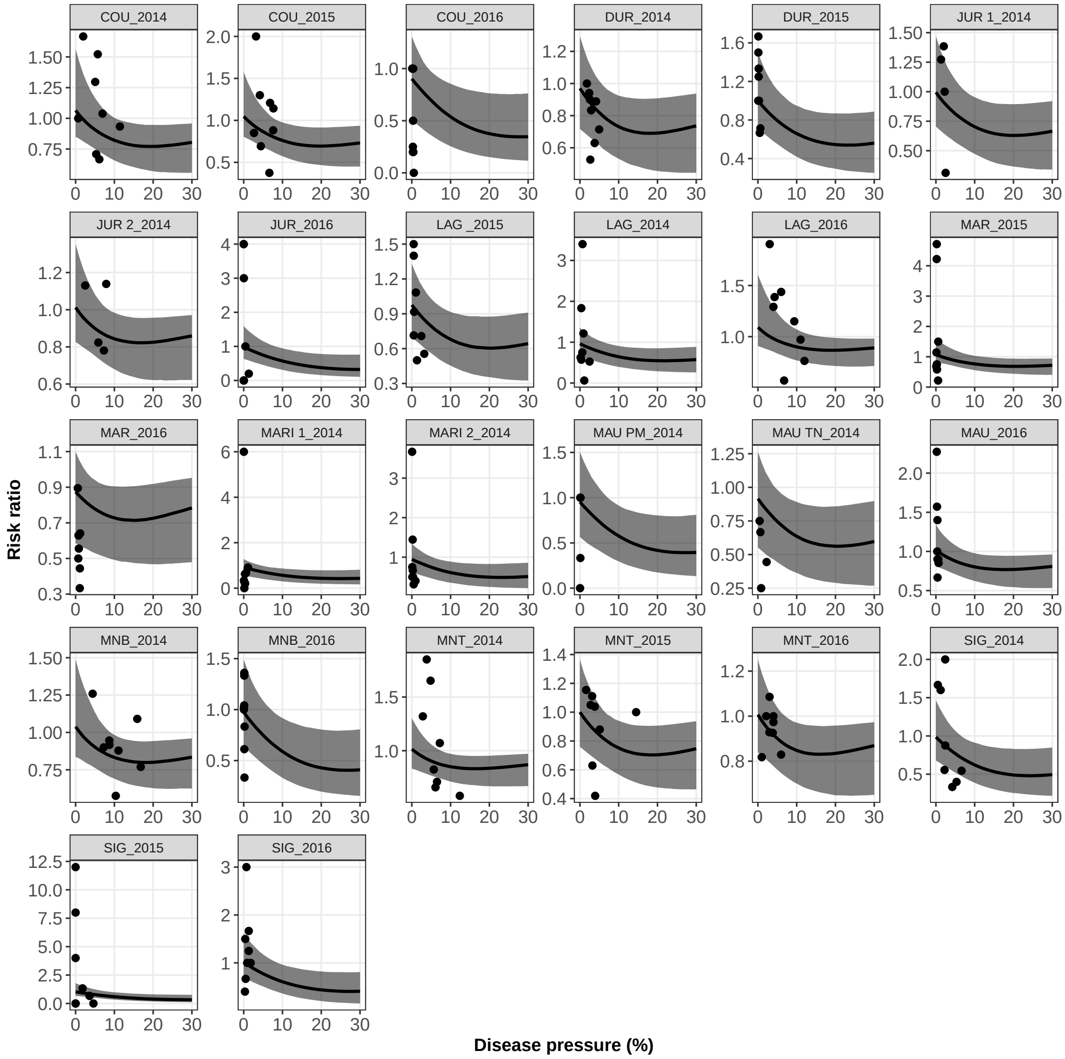

Figure 4: Individual risk ratios with *Aureobasidium pullulans* (black line) with their 95% credible bands (grey shadow) as a function of the intensity at the control. Observed risk ratio are represented by black dots."

Models comparison

```
GLRM_0 <- brm(ndisbunches | vint(nbunches) ~ TRT  +
               (1 | Trial_ID/Bloc) +
               (1 | Trial_ID:TRT) + (1 | Trial_ID:TRT:Bloc),
           data = dataA, family = beta_binomial2,
           iter = 3000,
           cores = 4, seed = 1234, stanvars = stanvars,
           control = list(adapt_delta = 0.95))

GLRM_Int <- brm(ndisbunches | vint(nbunches) ~ TRT + CTR_Int +
               TRT:CTR_Int + (1 | Trial_ID/Bloc) +
               (1 | Trial_ID:TRT) + (1 | Trial_ID:TRT:Bloc),
           data = dataA, family = beta_binomial2,
           cores = 4, seed = 1234, stanvars = stanvars,
           control = list(adapt_delta = 0.90))
```

```r
GLRM_3 <- brm(ndisbunches | trials(nbunches) ~ TRT + CTR_Int +
                         TRT:CTR_Int + (1 | Trial_ID/Bloc) +
                         (1 | Trial_ID:TRT) + (1 | Trial_ID:TRT:Bloc),
                       data = dataA, family = binomial,
                       cores = 4, seed = 1234,
                       control = list(adapt_delta = 0.9))

GLRM_4 <- brm(ndisbunches | vint(nbunches) ~ TRT + CTR_Int +
             TRT:CTR_Int + (1 | Trial_ID/Bloc),
           data = dataA, family = beta_binomial2,
           cores = 4, seed = 1234, stanvars = stanvars,
           control = list(adapt_delta = 0.90))

GLRM_5 <- brm(ndisbunches | vint(nbunches) ~ TRT + CTR_Int +
             TRT:CTR_Int + (1 | Trial_ID/Bloc) +
            (1 | Trial_ID:TRT) ,
           data = dataA, family = beta_binomial2,
           cores = 4, seed = 1234, stanvars = stanvars,
           control = list(adapt_delta = 0.90))
```

GLRM_0 and GLRM_Int are the models used in our manuscript to analyze the datasets.
GLRM_3 is similiar to GLRM_Int but with a binomial family, meaning the overdispersion is not taking into account.
GLRM_4 has no random interactions (i.e. (1 | Trial_ID:TRT) and (1 | Trial_ID:TRT:Bloc) are missing).
GLRM_5 is similar to GLRM_Int but with no random interaction between the block nested with trial and the treatment (i.e. (1 | Trial_ID:TRT:Bloc) is missing).

Table 3: Efficient approximate leave-one-out cross-validation for potassium bicarbonate.

model	elpd_diff	se_diff	WAIC
GLRM_Int	0.0	0.0	1935
GLRM_5	-4.3	4.6	1946
GLRM_4	-23.4	8.6	1986
GLRM_0	-42.9	11.1	2023
GLRM_3	-105.3	22.9	2134

Table 4: Efficient approximate leave-one-out cross-validation for Aureobasidium pullulans.

model	elpd_diff	se_diff	WAIC
GLRM_Int	0.0	0.0	2249
GLRM_5	-5.7	4.7	2262
GLRM_4	-11.3	8.0	2275
GLRM_0	-12.6	6.6	2275
GLRM_3	-122.7	29.6	2481

We compared the five fitted models (see Tables 3 and 4), for each biocontrol agent, by estimating the difference in their expected predictive accuracy by the difference in the expected log predictive density (elpd_loo).
A negative elpd_diff favors the first model.
We also computed the Widely Applicable Information Criterion (WAIC). A lower WAIC indicates a better fit.
