# Supplementary material for: On-Farm Trials Reveal Significant but Uncertain Control of Botrytis cinerea by Aureobasidium pullulans and Potassium Bicarbonate in Organic Grapevines
Source: Front Plant Sci. 2021 Feb 24;12:620786. doi: 10.3389/fpls.2021.620786 (PMC7943639; doi:10.3389/fpls.2021.620786)
Supplement: Supplementary file 2 [file Table_1.DOCX]

**On-farm trials reveal significant but uncertain control of *Botrytis cinerea* by *Aureobasidium pullulans* and potassium bicarbonate in organic grapevines.**

**Supplementary Material**

Anabelle Laurent, David Makowski, Nicolas Aveline, Séverine Dupin, Fernando Miguez

Table 1: Summary of previous published studies evaluating the efficacy (on incidence and severity) of biopesticides to control *Botrytis cinerea* on grapevine based on outdoor experiments.

| Reference | Experiment | Active ingredient(s) | Efficacy | Comments |
| --- | --- | --- | --- | --- |
| Pertot et al., 2017b | - field trials (commercial vineyards) in 3 locations in Italy for 4 years. - RCBD with 3 or 4 reps (minimum 8 vines per rep) - 20 bunches/rep for assessing symptoms | - ***Trichoderma atroviride*** applied at ‘berries beginning to touch’ stage - ***Aureobasidium pullulans*** *(Botector®)* applied at the ‘beginning of ripening’ stage - ***Bacillus subtilis*** applied 20 and 7 days before harvesting - ***T.atrovide+B.subtilis+ A.pullulans*** (combined strategy) | - mean Ei*: 75% to 85% - mean Es*: 85% to 95% - efficacy with single biopesticide comparable to combined strategy | - high level of efficacy can be explained with the relatively low-medium level of the disease - biopesticide applied at the specific stage fully controlled the disease (they survived until harvest at concentrations sufficient to prevent *B. cinerea* |
| Rotolo et al., 2018 | - two field trials conducted in two table grape vineyards - RCBD with 4 reps. One plot = 12 vines - 11 sprays (off-label spray numbers for research purpose) - 150-200 bunches/plot for assessing symptoms | - ***Bacillus subtilis*** - ***Bacillus amyloliquefaciens*** - ***Aureobasidium pullulans*** (Botector®) | - mean Ei ranged from 7.6 to 23% - mean Es ranged from 19% to 50% | - biopesticides not effective (<30%) under high disease pressure compared to alternate use of biopesticide with chemical fungicide |
| Calvo-Garrido et al., 2019 | - 4 vineyard sites between 2015 to 2017 - leaf removal in 2016 and 2017 only - RCBD 4 or 5 reps - two different application strategies: “full season” strategy = 5 or 6 applications and “late season”= after veraison and following determination of a Disease Risk Index | - ***Bacillus subtilis QST713*** - ***Bacillus amyloliquefaciens*** - ***Aureobasidium pullulans*** (Botector®) - ***Ulicladium oudemansii*** - ***Bacillus subtilis*** **IAB/BS03** | - Es for *B. amyloliquefaciens* ranged from 37 to 58% - Es for *A.pullulans* ranged from -24 to 48% - Es for *B. subtilis QST713* ranged from -43 to 54% - Es for *B. subtilis IAB/BS03* ranged from -17 to 25% - Es for U*. oudemansii* ranged from -59 to 47% | - pullulans significantly reduced intensity in one out of 4 sites-years - *B. subtilis QST713* significantly reduced the intensity in 4 out of 6 sites-years - *B. amyloliquefaciens* significantly reduced the intensity in 3 out of 5 site-years |
| Aziz et al., 2016 | - 2 vineyards in 2006 in France - canopy management was applied - RCBD with 12 plants per plot and 3 reps - intensity and incidence were performed on 50-60 clusters - treatments were applied twice in July (individually and in mixture) | - ***Bacillus subtilis*** - ***Pseudomonas fluorescens*** - ***Pantoea agglomerans*** - ***P. fluorescens + P. agglomerans*** - ***P. fluorescens + B. subtilis*** - ***P. agglomerans + B. subtilis*** | - Ei and Es for *P. fluorescens* were equal to 40.3% and 78%, respectively - Ei and Es for *P. agglomerans* were equal to 18.2% and 72%, respectively - Ei and Es for *B. subtilis* were equal to 43.6% and 87%, respectively - Ei ranged from 53.1% to 67.6% for mixtures - Es ranged from 78% to 93% for mixtures | - combination of two bacteria which were least effective when used alone (*P.fluorescens* and *P.agglomerans*) provided the best protection |
| Elmer et al., 2005 | - 2 vineyards over three growing season - RCBD with 4 or 6 reps (depending on the year) - treatment applied at 5% and 90% flowering | - ***Ulocladium oudemansii*** (BOTRY-Zen®) | - efficacy was equal to 92%,83% and 81% in 2003, 2004 and 2005, respectively (Ei or Es was not mentioned) |  |
| Reglinski et al., 2005 | - one field trial (commercial vineyard) in New Zealand - RBD - 10 bunches/vines and 5 vines/treatment | - ***Ulocladium oudemansii*** | - Ei = 78% | - *U. oudemansii* aggressively suppressed the growth and development of *B. cinerea* on necrotic tissue |
| O’Neill et al., 1996 | - 133 field experiments in 9 commercial vineyards (19 countries) between 1988 and 1994. - RBD with 4-6 reps - 3 to 30 plants per plot - 4 applications (end of flowering, closure bunches, veraison and 2-3 weeks after veraison) - incidence assessed on 50-100 bunches/plot | - ***Trichoderma harzianum*** T39 | - on average, Ei = 36 +/- 2.7% - average disease incidence in the untreated plots of all experiments = 42 +/- 2.3% | - if disease incidence in untreated plot>60%, biopesticide resulted in a 20% reduction |
| Magnin-Robert et al., 2013 | - research vineyard treated over 3 years. - RCBD with 5-12 plants/plot and 3 reps - measurement performed with 100 clusters/treatment - biopesticide applied in mixture only | - ***Acinetobacter lwoffii*** (two strains)= AL - ***B. subtilis*** (1 strain)= Bs - ***P. agglomerans*** (two strains)= PA - ***P. fluorescens*** (two strains)= PF | - treatments with mixtures of both strains of P*. agglomerans* had a leaf protection** equal to 53% - treatments combining *Acineobacter* spp. and *Pseudomonas* spp. = poor leaf protection (AL+PF, AL+Bs+PF, or AL+PA+PF+Bs) - treatments combining AL+PA+PF and all other combinations strongly protected the leaves: from 45 to 75% | - treatments with bacterial mixtures were shown to induce systemic resistance against *B. cinerea* in the second year - efficacy and duration of such a disease control seemed to be reinforced on year three without renewal of bacterial treatments |
| Cañamás et al., 2011 | - field trials in 2005 and 2006 (Spain) - RCBD with 4 reps - 5-7 vines per replication - 5 sprays: flowering, pea sized, veraison, 21 days before harvest and 7 days before harvest | - ***Candida sake*** CPA-1 (trying different formulations) - one fungicide | - Ei for all *C. sake* treatments ranged between 36% and 40% | - no significant difference between the *C. sake* treatments and the conventional fungicide in field trials |
| Youssef and Roberto, 2014 | - field trials located in Brazil - RCBD with 3 reps - 3 vines per treatment - Application one week before harvest | - **Potassium sorbate** - **Potassium bicarbonate** - **Calcium chelate** | - Ei ranged between 77% to 100% compared to a water control | - Potassium bicarbonate and potassium sorbate were the most effective |

*Ei = efficacy based on incidence (percentage of bunches with *B. cinerea* rot symptoms) ; Es = efficacy based on severity (percentage of *B. cinerea* rotten berries per bunch)

RBD= randomized block design ; RCBD = randomized complete block design

** leaf protection = reduction of the lesion size relative to the control (%)
